# Rapid flow assessment of congenital heart disease using high spatio-temporal gated spiral phase-contrast MR

**DOI:** 10.1186/1532-429X-13-S1-P380

**Published:** 2011-02-02

**Authors:** Jennifer A Steeden, David Atkinson, Andrew M Taylor, Vivek Muthurangu

**Affiliations:** 1 University College London, London, UK; 2 UCL, Institute of Child Health, London, UK

## Purpose

Many sick adults and children are unable to perform long breath-holds required for conventional, Cartesian phase-contrast (PC) sequences. Using a prospectively-triggered spiral PCMR sequence accelerated with sensitivity encoding (SENSE), it should be possible to achieve high resolution PCMR data in a short breath-hold.

The aim of this study was to compare flow volumes measured using: a) reference free-breathing, gated Cartesian PCMR, b) standard breath-hold, gated, Cartesian PCMR, and c)  gated, spiral, SENSE, breath-hold PCMR.

## Methods

40 consecutive children and adults were enrolled in this study (M:22, F:18, age:21.4±13.8 years). Flow was measured in the:

- Ascending aorta (AAO, N=40)

- Main pulmonary artery (MPA, N=38)

- Right pulmonary artery (RPA, N=22)

- Left pulmonary artery (LPA, N=24)

Flow assessment was performed in each vessel using the three sequences above (parameters shown in Table 1).

**Table 1:** 

	Free-Breathing PCMR	Standard Breath-hold PCMR	Spiral Breath-hold PCMR
TE/TR (ms)	~2.2 / 7.0	~2.2 / 7.0	2.1 / 8.0
Spiral Readouts	-	-	36
Acceleration factor	2 (GRAPPA)	2 (GRAPPA)	3 (SENSE)
Matrix Size	256 x 192	192 x 113	256 x 256
FOV (mm)	200 - 400	290 - 400	400
Rectangular FOV (%)	75	66	100
Readouts per segment	3	4	2
Pixel bandwidth (Hz/pixel)	543	543	1220
VENC (cm/s)	180 - 400	180 - 400	180 - 400
NSAs	3	1	1
Gating	Retrospective	Retrospective	Prospective
Total Scan Duration (s)	44 - 144	11 - 24	3 - 8
Voxel Size (mm)	~ 0.8 - 1.5	~ 1.5 - 2.1	1.6
Temporal resolution	~ 30.0 ms	~ 40.0 ms	32.0 ms

Stroke volume and regurgitation fraction were calculated for each patient. Additionally, Qp/Qs (N=38) and RPA/LPA (N=20) ratios were quantified where possible.

## Results

Average scan time was 91±17 seconds for the reference free-breathing sequence, 16±3 seconds for the standard breath-hold sequence, and 5±1 seconds for the spiral breath-hold sequence.

**Figure 1:**
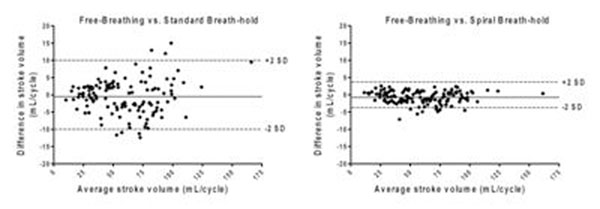


Combining all vessels (N=124), there were no statistical differences in mean stroke volume calculated from the reference free-breathing sequence (60.3±27.3 mL), the standard breath-hold sequence (59.8±27.6 mL) and the spiral breath-hold sequence (59.5±27.1 mL). Bland-Altman analyses are shown in Figure 1. There was no clinically significant bias using either breath-hold sequence (spiral breath-hold: -0.7 mL, standard breath-hold: -0.5 mL). However, the limits of agreement were smaller and the correlation better for the spiral breath-hold compared to the standard breath-hold sequence (-4.4 to 2.9 mL vs. -10.3 to 9.3 mL, respectively).

There was also an excellent agreement in QP/QS and RPA/LPA ratios between all sequences, however the spiral breath-hold sequence was found to be superior to the standard breath-hold sequence in terms of limits of agreement and correlation. There was a small but statistically significant underestimation of regurgitation fraction using the spiral sequence.

## Conclusion

Flow volumes can be accurately and reliably quantified using a spiral SENSE PCMR sequence, with high spatio-temporal resolution in a short breath-hold. As the standard method of measuring flow in congenital heart disease is free-breathing, cardiac gated PCMR, this spiral sequence could reduce total flow imaging from ~10 minutes, to <1 minute. This is a marked reduction in total scan time and has implications for patient throughput and compliance for congenital cardiac MR scanning.

